# Aqueous Cinnamon Extract (ACE-*c*) from the bark of *Cinnamomum cassia *causes apoptosis in human cervical cancer cell line (SiHa) through loss of mitochondrial membrane potential

**DOI:** 10.1186/1471-2407-10-210

**Published:** 2010-05-18

**Authors:** Soumya J Koppikar, Amit S Choudhari, Snehal A Suryavanshi, Shweta Kumari, Samit Chattopadhyay, Ruchika Kaul-Ghanekar

**Affiliations:** 1 Interactive Research School for Health Affairs (IRSHA), Bharati Vidyapeeth University Medical College Campus, Pune, Maharashtra, India; 2 National Center for Cell Science (NCCS), Pune University Campus, Ganeshkhind, Pune, Maharashtra, India

## Abstract

**Background:**

Chemoprevention, which includes the use of synthetic or natural agents (alone or in combination) to block the development of cancer in human beings, is an extremely promising strategy for cancer prevention. Cinnamon is one of the most widely used herbal medicines with diverse biological activities including anti-tumor activity. In the present study, we have reported the anti-neoplastic activity of cinnamon in cervical cancer cell line, SiHa.

**Methods:**

The aqueous cinnamon extract (ACE-*c*) was analyzed for its cinnamaldehyde content by HPTLC analysis. The polyphenol content of ACE-*c *was measured by Folin-Ciocalteau method. Cytotoxicity analysis was performed by MTT assay. We studied the effect of cinnamon on growth kinetics by performing growth curve, colony formation and soft agar assays. The cells treated with ACE-*c *were analyzed for wound healing assay as well as for matrix metalloproteinase-2 (MMP-2) expression at mRNA and protein level by RT-PCR and zymography, respectively. Her-2 protein expression was analyzed in the control and ACE-*c *treated samples by immunoblotting as well as confocal microscopy. Apoptosis studies and calcium signaling assays were analyzed by FACS. Loss of mitochondrial membrane potential (Δψ_m_) in cinnamon treated cells was studied by JC-1 staining and analyzed by confocal microscopy as well as FACS.

**Results:**

Cinnamon alters the growth kinetics of SiHa cells in a dose-dependent manner. Cells treated with ACE-*c *exhibited reduced number of colonies compared to the control cells. The treated cells exhibited reduced migration potential that could be explained due to downregulation of MMP-2 expression. Interestingly, the expression of Her-2 oncoprotein was significantly reduced in the presence of ACE-*c*. Cinnamon extract induced apoptosis in the cervical cancer cells through increase in intracellular calcium signaling as well as loss of mitochondrial membrane potential.

**Conclusion:**

Cinnamon could be used as a potent chemopreventive drug in cervical cancer.

## Background

Cervical cancer, which accounts for the second most common malignancy among women worldwide, is highly radio-resistant, often resulting in local treatment failure [1]. For locally advanced disease, radiation is combined with low-dose chemotherapy; however, this modality often leads to severe toxicity. Complementary and Alternative Medicine (CAM) is recently becoming a popular treatment for various cancers among which herbal medicine is one of the methods used in cancer therapy [2,3]. Currently, plants, vegetables, herbs and spices used in folk and traditional medicine have been accepted as one of the main sources of chemopreventive drugs [4-8]. Traditional medicine that includes herbal medicine has been used from time immemorial to treat chronic ailments such as cancer. Recently, scientific studies support herbal medicine as potent anti-cancer drug candidates [9-13].

Cinnamon, a widely used food spice, has been shown to exhibit diverse biological functions including anti-inflammatory [14], anti-oxidant [15,16], anti-microbial [15,17], and anti-diabetic effects [18-20]. Recently, the anti-tumor activity of cinnamon has been shown both in vitro [21-23] and *in vivo *[10]. Cinnamaldehyde, the bioactive component of cinnamon, has been shown to inhibit proliferation of several human cancer cell lines including breast, leukemia, ovarian, and lung tumor cells [24]. Recently, we reported a comparative analysis of cytotoxic effect of aqueous extract of cinnamon (ACE) from *C. zeylanicum *with that of commercial cinnamaldehyde on a variety of cell lines [23]. Compared to the commercial cinnamaldehyde, ACE proved to be more cytotoxic owing to the presence of polyphenolic compounds, besides cinnamaldehyde, that may act synergistically to induce enhanced cytotoxicity.

In the present work, we have reported the putative mechanism of cancer cell growth inhibition by aqueous cinnamon extract (ACE-*c*), from the bark of *Cinnamomum cassia *L. family Lauraceae, in a human cervical cancer cell line, SiHa. We observed that cinnamon altered the growth kinetics of cells in a dose-dependent manner. Our colony formation and soft agar assays demonstrated that the number of colonies in cells treated with ACE-*c *was less compared to the untreated control cells. The ACE-*c *treated cells exhibited slow migration potential compared to the control cells that could be explained due to reduced MMP-2 expression in the former. Cinnamon extract increased the intracellular calcium that might be responsible for the loss of mitochondrial membrane potential (Δψ_m_), finally leading to cellular apoptosis.

## Methods

### Reagents

Tissue culture plasticware was purchased from BD Biosciences, CA, USA; Axygen Scientific Inc, CA, USA and Nunc, Roskilde, Denmark. Dulbecco's Modified Eagles Medium (DMEM) was obtained from Himedia Corporation, Mumbai, India. Penicillin and streptomycin were obtained from Gibco BRL, CA, USA. Fetal bovine serum was purchased from Moregate Biotech, Australia, N. Z and 3-(4,5-dimethylthiazol-2-yl)-2,5-diphenylthiazolium bromide (MTT), FCCP, JC-1 and TMRE were purchased from Sigma-Aldrich (St. Louis, MO). Her-2 antibody was purchased from Santa Cruz Biotechnology, CA, USA, Donkey anti-Mouse IgG Cy-3conjugate (Millipore, MA) and Annexin V-FITC apoptosis kit #3 from Invitrogen (CA, USA). All other common reagents were procured from Qualigens fine chemicals (Mumbai, India).

### ACE-c preparation and characterization

The bark of *Cinnamomum cassia *was purchased from Shivam Ayurvedics Pune, Maharashtra, India with voucher specimen number for *Cinnamomum cassia *bark was 104. The sample was authenticated from Regional Research Institute (AY) Kothrud, Pune (ref no.1045). The bark was weighed, powdered and extracted in double distilled water (the ratio of cinnamon: water used was 1:16) in a hot water extractor [25]. The resulting extract was centrifuged at 13000 rpm for 15 min to remove the particulate matter. The supernatant was further filter-sterilized using swiney filter (pore size, 0.45 μm) and the resultant filtrate was stored in aliquots at -80°C until use. The bark identity was further confirmed by detecting the marker molecule cinnamaldehyde in ACE-*c *by HPTLC analysis as described previously [23,26]. The total polyphenol content of ACE-c was measured by Folin-Ciocalteau method as described previously [23,26].

### Cell culture

The human cervical carcinoma cell line, SiHa, used in the study was obtained from National Centre for Cell Science (NCCS), Pune, India. The cells were grown in DMEM containing 2 mM L-glutamine supplemented with 10% fetal bovine serum and 100 U/ml of penicillin-streptomycin. The cells were incubated in a humidified 5% CO_2 _incubator at 37°C.

### Cell viability

The cell viability was determined by MTT dye uptake as described previously [23]. Briefly, SiHa cells were seeded at a density of 1 × 10^5 ^cells/ml density in 96-well plates. An untreated group was kept as a negative control. The aqueous cinnamon extract (ACE-*c*) was added at following concentrations: 10, 20, 40, 80, 160 and 320 μg/ml, in wells in triplicates. The MTT solution (5 mg/ml) was added to each well, and the cells were cultured for another 4 h at 37°C in 5% CO_2 _incubator. The formazan crystals formed were dissolved by addition of 90 μl of SDS-DMF (20% SDS in 50% DMF). After 15 min, the amount of colored formazan derivative was determined by measuring optical density (OD) using the ELISA microplate reader (Biorad, Hercules, CA) at 570 nm (OD_570-630 nm_). The percentage viability was calculated as: % Viability = [OD of treated cells/OD of control cells] × 100

### Cell growth analysis

SiHa cells were seeded at a density of 1 × 10^5 ^cells/ml in 24-well plates in triplicates. Next day, the cells were dosed with different concentrations of ACE-*c *(0, 10, 20, 40 and 80 μg/ml) and grown for 24, 48 and 72 h. The cells were harvested and counted for viability using trypan blue dye exclusion method.

### Colony formation assay

The cells were plated at a seeding density of 1 × 10^3 ^cells/ml in 6-well plates. After 24 h, the cells were exposed to various concentrations of ACE-*c*: 0, 10, 20, 40, and 80 μg/ml. Plates were incubated at 37°C in a 5% CO_2 _incubator for one week. This was followed by fixing the colonies with 4% paraformaldehyde and staining with 0.5% crystal violet [27]. The colonies were photographed with Sony DSC-S75 cyber-shot camera.

### Soft agar assay

Control SiHa cells (5 × 10^3 ^cells/ml) as well as cells treated with different concentrations of ACE-*c *(10-80 μg/ml) were mixed at 40°C with 0.35% agarose (DNA grade, GIBCO BRL, CA, USA) in culture medium and gelled at room temperature for 20 min over a previously gelled layer of 0.5% agarose in culture medium in 6-well plates. After incubation for 10 days, colonies were photographed directly using an Axiovert 200 M microscope (Carl Zeiss, Germany) and counted [27].

### Wound healing assay

Cells were plated at a seeding density of 4 × 10^5 ^cells/ml in 24-well plates and grown overnight at 37°C in 5% CO_2 _incubator. An artificial wound was made with 10 μl micropipette after 6 h serum starvation in control cells as well as cells treated with different concentrations of ACE-*c *(10-80 μg/ml). Time-lapse imaging of migrating cells was performed on Nikon Eclipse TE2000-E microscope (Nikon, Tokyo, Japan) over 15 h in serum containing medium in a humidified chamber at 37°C and 5% CO_2 _atmosphere. Images were obtained every 20 min using a 10× phase objective of NA 0.25 and analyzed by image analysis software Metamorph Universal Imaging, USA. The average migration rate in μm/h was calculated and graphs were plotted using Microsoft Excel and Sigma plot program.

### RT-PCR analysis

The total cellular RNA from control as well as cells treated with different doses of ACE-*c *(0-80 μg/ml) was extracted by a one-step acid guanidine isothiocyanate-phenol method using TRI reagent (Sigma, St. Louis, MO). RNA was precipitated with isopropanol and the concentration was estimated by spectrophotometer (Biorad, SmartSpec™ 3000). Ten microgram of total RNA was used for each RT-PCR reaction. Fifty units of Moloney murine leukemia virus reverse transcriptase (MMuLV) (Bangalore Genei, Banglore, India) were added in a typical 50 μl reaction (10 μg RNA, 1× first-strand buffer, 1 mM DTT, 2.5 mM dNTPs, 50 ng/μl random primers and 15 U/μl RNAse i) and incubated for 1 h at 42°C followed by incubation at 95°C for 5 min. The purified cDNA template was amplified using different sets of primers. The primers used were as follows: β-actin-F: 5'-taccactggcatcgtgatggact-3'; β-actin-R: 5'-tttctgcatcctgtcggaaat-3'; MMP-2-F: 5'-ggctggtcagtggcttggggta-3'; MMP-2-R: 5'-agatcttcttcttcaaggaccggtt-3'. PCRs were performed in 25 μl volume in which 1× PCR buffer, 2.5 mM dNTPs, 1.5 mM MgCl_2_, 1 U of Taq polymerase and 100 ng of the specific primers were added. A brief initial denaturation at 95°C for 5 min was followed by 35 cycles with the following steps: 95°C for 1 min, annealing at 55-55.2°C for 1 min and extension at 72°C for 1 min. RT-PCR products were then separated on a 1.2% agarose gel and visualized by staining with ethidium bromide. The intensities of the bands corresponding to the RT-PCR products were quantified using phosphorimager (Alpha Imager using Alpha Ease FC software, Alpha Innotech) and normalized with respect to the β-actin product.

### Gelatin zymography

The Gelatin zymography was performed to detect the presence of extracellular MMP-2 [28]. The conditioned medium of control cells as well as cells treated with 80 μg/ml ACE-*c *was collected and concentrated in Centricon YM-30 tubes (Millipore, MA). Both the control as well as the treated samples containing equal amount of total proteins were mixed with sample buffer (2% SDS, 25% glycerol, 0.1% bromophenol blue and 60 mM Tris- HCl pH 6.8) and loaded onto 7.5% SDS-polyacrylamide gel containing gelatin (0.5 mg/ml). The gel was washed with 0.25% Triton X-100 and incubated overnight in incubation buffer (150 mM NaCl, 100 mM CaCl_2_, 50 mM Tris-HCl pH 7.5, 1% Triton X- 100, 0.02% NaN_3_) at 37°C. The gel was stained with staining solution (0.1% Coomassie Brilliant blue R-250 in 40% isopropanol) and destained in 7% acetic acid. Gelatinolytic activity was detected as unstained bands on a blue background. The quantitation of bands in control and treated samples was performed by densitometric analysis on Alpha Imager using Alpha Ease FC software, Alpha Innotech.

### Immunoblotting

Cell extracts were prepared from control as well as cells treated with different concentrations of ACE-*c*: (0-80 μg/ml). Briefly, the cell pellet was resuspended in 80 μl lysis buffer containing 50 mM Tris (pH 7.4), 5 mM EDTA, 0.5% NP40, 50 mM NaF, 1 mM DTT, 0.1 mM PMSF, 0.5 μg/ml leupeptin (Pro-pure Amersco, Solon, USA), 1 μg/ml pepstatin (Amresco, Solon, USA), 150 mM NaCl, 0.5 μg/ml aprotinin (Amersco, Solon, USA) and protease inhibitor cocktail (Roche, Lewes, UK) and incubated on ice for 1 h with intermittent mixing. The extract was centrifuged for 20 min at 4°C at 12000 rpm. The protein was estimated using Bradford reagent (Biorad Laboratories Inc, CA, USA). Equal amount of protein was loaded on a 10% SDS-polyacrylamide gel and transferred electrophoretically to Amersham Hybond-P PVDF membrane (GE Healthcare, UK) in sodium phosphate buffer (pH 6.8). The membrane was blocked in 5% BSA in TST and incubated at room temperature for 1 h with mouse monoclonal antibody for Her-2 and tubulin (Santacruz, CA, USA) at a 1:1000 and 1:2500 dilution, respectively. The membrane was washed in TST and incubated with donkey anti mouse IgG HRP conjugate at 1:5000 (for Her-2) and 1:3000 (for tubulin) dilutions. Proteins were visualized using a chemiluminescence kit (Amersham ECL Advance western blotting detection kit, GE Healthcare, UK) and densitometric analysis of X-ray films was performed on Alpha Imager using Alpha Ease FC software, Alpha Innotech.

### Measurement of Apoptosis

The cells were plated at a seeding density of 5 × 10^5 ^cells/well and treated with different concentrations of ACE-*c*: (0-80 μg/ml). After 24 h of treatment, the cells were harvested and washed with PBS twice. Cells were stained with Annexin V-FITC following the manufacturer's instructions (Annexin V-FITC apoptosis kit #3, Invitrogen) and analyzed for apoptosis by FACS using CellQuest Software.

### Intracellular calcium measurement by flow cytometry

Intracellular Ca^2+ ^levels were analyzed in control cells as well as cells treated with different doses of ACE-*c*: (0-80 μg/ml) by flow cytometry [29]. Cells were loaded with 5 μM Fluo-3/AM (Sigma, St. Louis, MO) and 100 μg/ml of Pluronic F127 (Sigma, St. Louis, MO) in centrifuge tubes and incubated at 37°C, 5% CO_2 _for 1 h in the dark. The cells were resuspended after every 20 min to ensure even dye loading. The cell pellets were washed twice with 0.9% saline and finally resuspended in 3 ml Hank's Balanced Salt Solution (HBSS) in FACS tubes. Ionomycin (30 μM) was used as a positive control. Fluorescence intensities were measured at 525 nm by FACS Calibur (Becton Dickinson Immunocytometry Systems, San Jose, CA) to obtain baseline readings. Mean channel fluorescence intensities were calculated using CellQuest software.

### Detection of Mitochondrial Membrane Potential (Δψm) using JC-1

Mitochondrial membrane potential was estimated using 2.5 μg/ml JC-1 fluorescent dye either by confocal microscopy or by flow cytometry. For confocal studies, cells were seeded at a density of 1 × 10^5 ^cells/ml on coverslips in 6-well plates. After 24 h, cells were treated with different concentrations of ACE-*c*: (0-80 μg/ml). Next day, the media was removed and the cells were incubated with fresh culture medium containing JC-1 dye for 30 min at 37°C in the dark. Cells on coverslips were washed with PBS twice and fixed with 2.5% paraformaldehyde made in 200 mM HEPES buffer for 15 min at room temperature followed by PBS wash. The coveslips were mounted in antifade mounting medium containing DAPI (Ultracruz mounting medium, Santacruz) on glass slides. The cells were then analyzed for JC-1 uptake by using Zeiss LSM510 META confocal laser scanning microscope (Zeiss, Thornwood, NY) having LSM Image Examine software. For *Δψm *detection by flow cytometry [30], 5 × 10^5 ^cells were plated in 6-well plates. The cells were treated with different concentrations of ACE-*c *(0-80 μg/ml). Twenty four hours post treatment; the cells were harvested, washed with PBS and incubated with culture medium containing JC-1 for 30 min at 37°C in the dark. Cells were washed in PBS twice and analyzed for *Δψm *using FACS. FCCP (10 μM) was used as a positive control. The fluorescence intensities were measured at 527 nm (green) and 590 nm (red). Analysis was done by CellQuest software.

### Immunofluorescence microscopy

For immunostaining SiHa cells were plated on coverslips in 6-well plates at a seeding density of 2 × 10^5 ^cells/ml. After 24 h, the cells were dosed with different concentrations of ACE-*c *(0-80 μg/ml). Twenty four hours post-treatment; the cells were washed with PBS and fixed in 2.5% paraformaldehyde made in 200 mM HEPES buffer for 15 min at room temperature. Cells were washed for 5 min in PBS, permeabilized with 0.1% Triton X-100 in PBS for 5 min and blocked in 10% FBS (made in PBS) for 1 h. For detection, the cells were incubated with Her-2 antibody (Santa Cruz Biotechnology, Santa Cruz, CA) that was diluted in blocking buffer at 1:100 dilution. After washing with PBS, the cells were incubated with the secondary antibody [CY3-conjugated antimouse immunoglobulin (Millipore)] at a dilution of 1:300. Slides were then mounted in antifade mounting medium (Ultracruz mounting medium, Santacruz) and analyzed with a Zeiss LSM 510 META confocal laser scanning microscope (Zeiss, Thornwood, NY) using LSM Image Examine software.

### Statistical analysis

All experiments were performed in triplicates and repeated at least five times and the data were presented as mean ± SD. Statistical analysis was conducted with the SigmaStat 3.5 program (Systat Software, Inc.) using one-way ANOVA. The α level used for comparisons was α = 0.05.

## Results

### Cinnamon treatment alters growth kinetics of SiHa cells

Aqueous extract of cinnamon prepared from *C. cassia *(ACE-*c*) was analyzed for the presence of cinnamaldehyde as well as polyphenols to ensure the quality and purity of the preparation [see Additional file 1: Figs. S1 A and S1 B]. We initially performed MTT assay to define the optimal concentration at which cinnamon was non-toxic to cells. Up till 0.32 μg/ml ACE-*c *concentration, the cells exhibited 100% survival within 24 h [see Additional file 1: Fig. S2].

**Figure 1:**
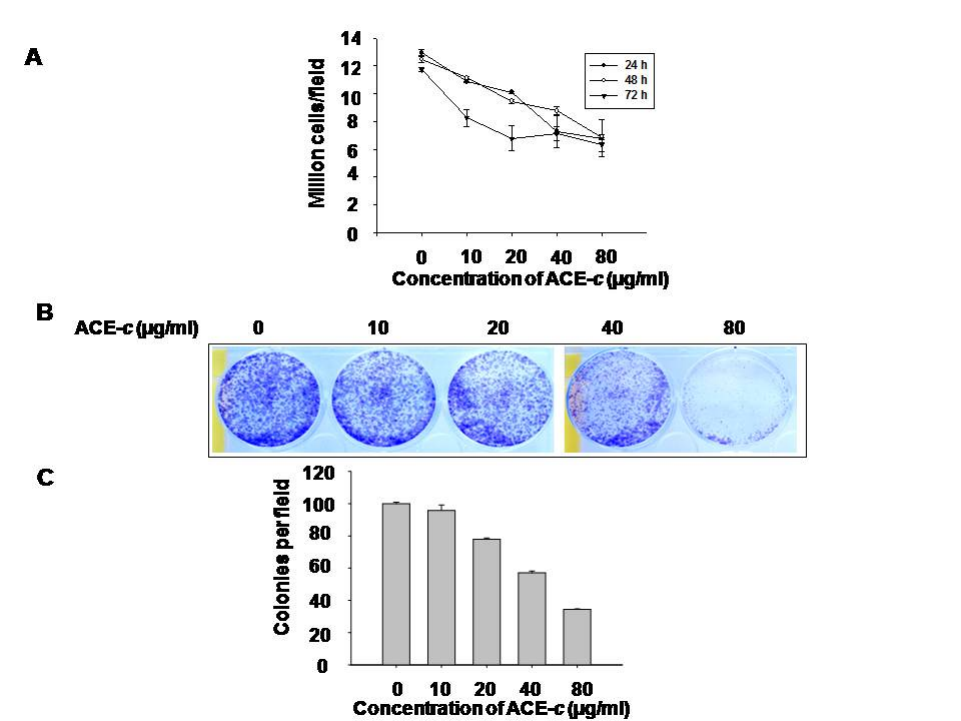
**Cinnamon alters growth kinetics of cervical cancer cells**. (A) The cells were treated with various concentrations (0-80 μg/ml) of ACE-*c *for 24, 48 and 72 h, and the number of viable cells were counted using the trypan blue dye exclusion assay. The growth kinetics has been presented in the figure taken at different time points. Data represent mean ± SD of five different experiments. (B) The cells (1 × 10^3^/ml) were grown in 6-well plates and treated with various concentrations (0-80 μg/ml) of ACE-*c *for one week. The cells were then stained with crystal violet and photographed. The experiments were repeated five times. (C) The cells (5 × 10^3^) were treated with various concentrations (0-80 μg/ml) of ACE-*c *and grown in soft agar for 10 days, and the colonies were counted. Colonies were counted from at least 10 different areas, and the average of each is plotted. The data represent mean ± SD of five independent experiments.

To test the effect of cinnamon on the growth kinetics, SiHa cells were treated with different concentrations of ACE-*c: *0, 10, 20, 40, and 80 μg/ml and were grown for 24, 48 and 72 h. At the end of each treatment, the cells were stained with trypan blue, and the viable cells that excluded the dye were counted. It was observed that there was a dose- dependent decrease in the growth kinetics of ACE-*c*-treated cells compared to the untreated control cells (Fig. 1A). Moreover, it was found that at around 80 μg/ml concentration of ACE-*c *treatment, there was a significant decrease (~2-fold) in the growth kinetics compared to that observed in the untreated control cells (p ≤ 0.05 for 24 h; p ≤ 0.001 for 48 h and 72 h). This was further confirmed by colony forming assay wherein at a lower seeding density, cells were treated with different concentrations of ACE-*c *for one week. At 80 μg/ml concentration of ACE-*c*, the cells exhibited relatively lesser colonies compared to the control cells (Fig. 1B). Consistent with the slow growth rate, it was observed that cinnamon extract induced a dose-dependent decrease in the number of soft agar colonies. Interestingly, at 80 μg/ml ACE-*c *treatment, the number of soft agar colonies was reduced by ~3-fold (*p *≤ 0.001) compared to the untreated control cells (Fig. 1C). All these data indicated that cinnamon altered the growth kinetics of SiHa cells in a significant manner that could be a positive indicator for testing its antineoplastic activity in cervical cancer cells.

### Cinnamon extract decreases cell migration through reduction in MMP-2 expression

**Figure 2:**
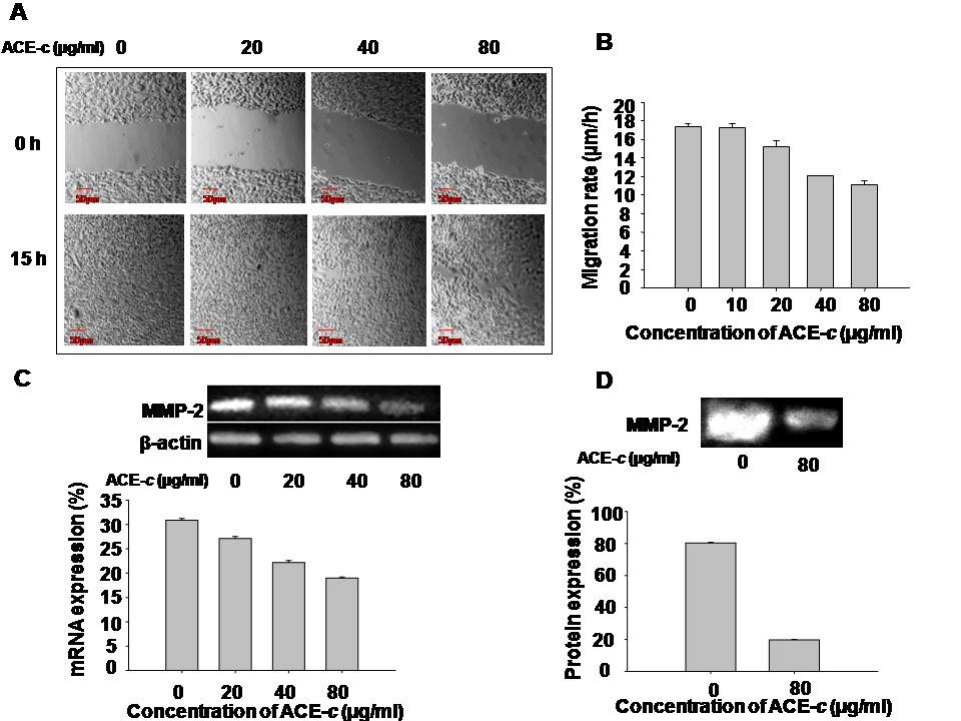
**Cinnamon reduces migration potential**.(A) Photomicrographs of time-lapse image at 0 and 15 h in a wound-healing assay in cells treated with different concentrations (0-80 μg/ml) of ACE-*c*. The upper panel of the image shows the wound made at 0 h. The lower panel shows cell movement corresponding to the distance traveled by the cells at 15 h of time-lapse imaging. (B) Rate of migration of cells during the wound healing assay analyzed by the time-lapse imaging of SiHa cells. Migration rate (μm/h) for each sample from five different fields was calculated. Error bars represent standard deviation and the data is representative of five independent experiments. (C) ACE-*c *treatment reduces the MMP-2 expression at mRNA level that has been shown by RT-PCR. β-actin was used as the loading control. Densitometric analysis of MMP-2 expression was performed using phosphorimager. The data represents mean ± SD of five independent experiments. (D) Gelatin zymography showing downregulation of MMP-2 expression in SiHa cells at 80 μg/ml ACE-*c *treatment compared to the untreated control cells. The bands were quantified by densitometry using phosphorimager and the data represents mean ± SD of five independent experiments.

To examine the effect of ACE-*c *on cell migration, we performed wound healing assay on confluent monolayers of SiHa cells. After making the wound with a pipette tip, the cells were cultured in presence or absence of different concentrations of the aqueous cinnamon extract and imaged with real time-lapse video for a period of 15 h. It was observed that ACE-*c *effectively inhibited the migration of cells in a dose- and time-dependent manner compared to the untreated control cells (Fig. 2A and 2B). The control cells filled-up the wound gap completely after 15 h whereas in cells treated with ACE-*c*, particularly at 40 and 80 μg/ml concentrations, the wound gap was not completely filled. Interestingly, at 80 μg/ml, cinnamon treatment significantly decreased (~1.5-fold; *p *≤ 0.001) the migration rate of SiHa cells thereby affecting their migration capability.

Since MMP-2 is known to play a significant role in the invasive property of tumor cells, we investigated the mechanism behind the delay in wound healing exhibited by ACE-*c *treated cells. We tested the expression of MMP-2 in cells treated with/without cinnamon extract. It was observed that the expression of MMP-2 was significantly down-regulated both at mRNA (Fig. 2C) as well as protein level (Fig. 2D) in a dose-dependent manner compared to the untreated control cells. Interestingly, at 80 μg/ml concentration of ACE-*c*, there was a ~1.6-fold (p ≤ 0.001) decrease in MMP-2 transcript and ~4-fold (p ≤ 0.001) down regulation in the expression of MMP-2 protein. These data suggested that ACE-*c *induced decrease in the migration of cervical cancer cells through down-regulation of MMP-2 expression.

### Cinnamon treatment downregulates the expression of Her-2 oncoprotein

**Figure 3:**
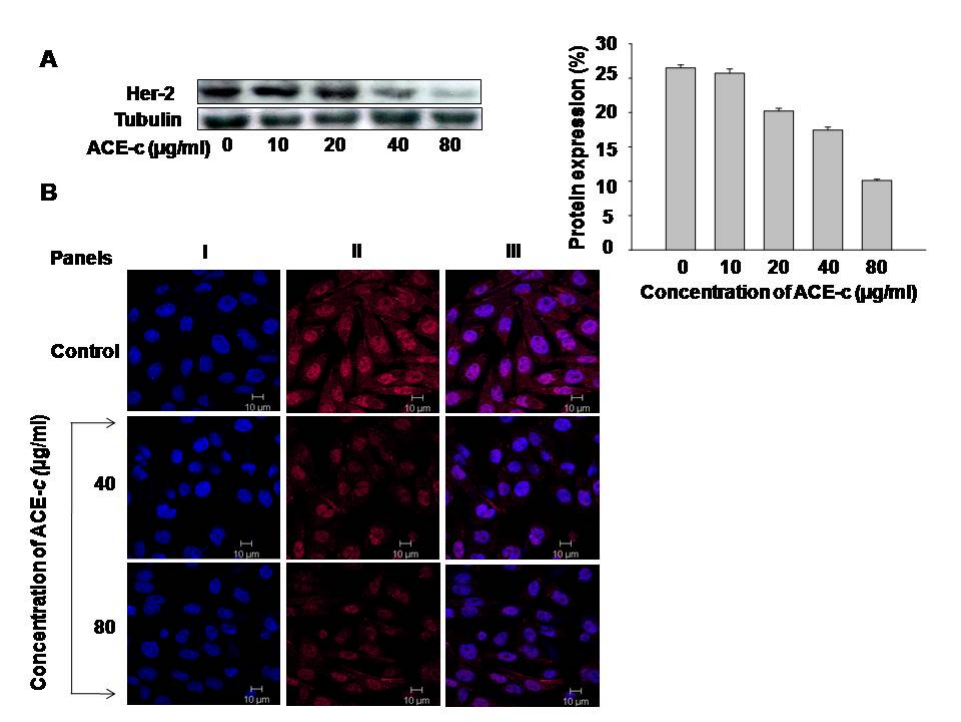
**Cinnamon decreased the expression of Her-2 oncoprotein**. (A) Western blot analysis showing decrease in Her-2 expression in SiHa cells treated with different concentrations of ACE-*c *(0-80 μg/ml). Equal amounts of protein were loaded on 10% SDS-gel and immunoblotted with anti-Her-2 antibody. Tubulin was used as a loading control. Densitometric analysis of Her-2 expression was performed using phosphorimager. The data represents mean ± SD of five independent experiments. (B) Confocal images of the cells treated with indicated concentrations of ACE-*c *showing decrease in Her-2 expression. The cells were stained indirectly for Her-2 using Cy3 antibody (Panel II) and counterstained with DAPI (Panel I). Panel III represents the merge images.

Various studies have shown that a variable proportion of cervical carcinoma tumors overexpress Her-2 oncoprotein [31]. Moreover, there are reports suggesting a correlation between Her-2 overexpression and upregulation of MMP-2 and MMP-9 expression [32,33]. Since we found that cinnamon downregulated the expression of MMP-2, we wanted to examine the status of Her-2 in cinnamon-treated cells and correlate its expression with that of MMP-2. Thus SiHa cells were treated with different concentrations of ACE-*c *(0-80 μg/ml) followed by immunoblotting of the extracted proteins using Her-2 antibody. Interestingly, we observed that the cinnamon extract down regulated the expression of Her-2 protein in a dose-dependent manner compared to the control cells (Fig. 3A), the maximum reduction being at 80 μg/ml (~2.6-fold, p ≤ 0.001). This was further proved by confocal studies wherein at 80 μg/ml of ACE-*c *treatment, a significant reduction in the expression of Her-2 could be observed (Fig. 3B). These results correlated with decreased MMP-2 expression observed in cinnamon-treated cells, thereby elucidating the potential antineoplastic role of cinnamon in cervical cancer through reduction of Her-2 and MMP-2 expression.

### Cinnamon extract induces apoptosis through increase in intracellular calcium as well as loss of mitochondrial membrane potential

**Figure 4:**
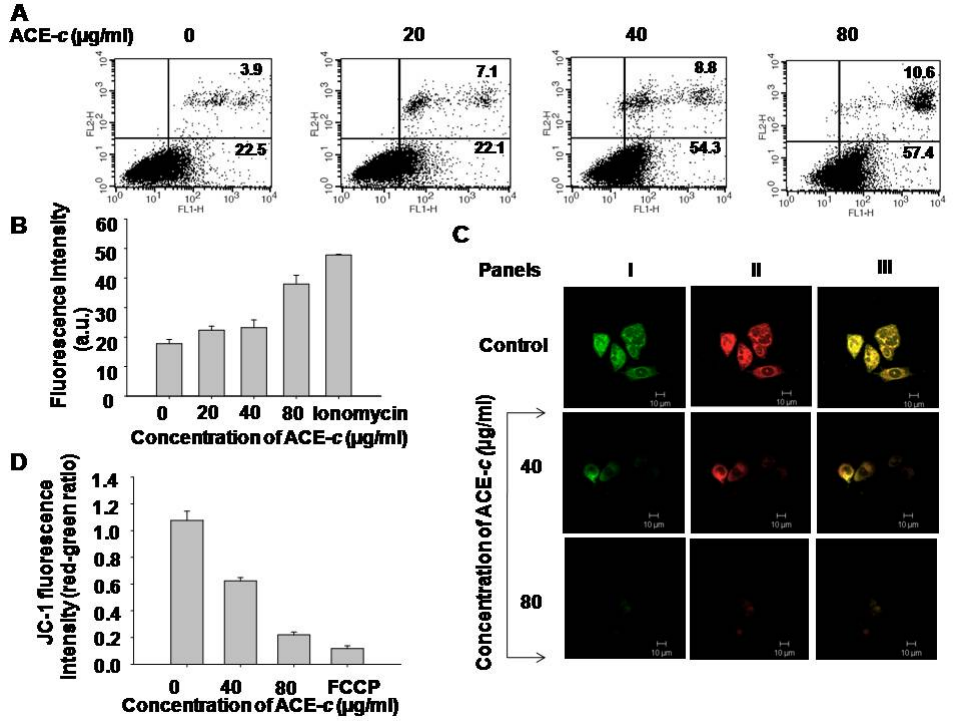
**Cinnamon induces apoptosis in SiHa cells through dysregulation of mitochondrial membrane potential**. (A) SiHa cells were treated with different concentrations of ACE-*c *(0-80 μg/ml) followed by Annexin V-FITC and PI staining to analyze the effect of cinnamon in apoptosis. This was determined by FACS analysis showing the percentage of early (lower right quadrant) and late (upper right quadrant) apoptotic cells. (B) Flow cytometric analysis of the rapid calcium release in SiHa cells after treatment with cinnamon. Cells (5 × 10^3 ^cells) were treated with different doses (0-80 μg/ml) of ACE-*c *for 24 h. This was followed by loading the cells with Fluo-3/AM for 1 h before analyzing in calcium-free HBSS. Ionomycin was used as a positive control. Fluorescence intensities were measured with FACS Calibur flowcytometer. The data represents mean ± SD of five independent experiments. (C) Confocal images showing mitochondrial membrane depolarization induced by cinnamon. Control and cinnamon-treated SiHa cells were stained with JC-1 and the staining pattern was monitored by confocal laser scanning microscopy. For detection of J-aggregate form (red) (Panel II) and J-monomer alone (green) (Panel I), Argon-Krypton laser line was excited at 590 nm and 527 nm, respectively. Panel III represents the merge images. (D) Flow cytometric analysis with JC-1 dye showing decrease in red to green fluorescence ratio. Control (5 × 10^5^) and cells treated with various concentrations (0-80 μg/ml) of ACE-*c *were stained with JC-1 dye for 30 min. Fluorescence intensities were measured with FACS Calibur flowcytometer. The data represents mean ± SD of five independent experiments.

To further elucidate the anti-cancer mechanism of cinnamon in cervical cancer cells, we performed apoptosis studies. After treating the cells with different doses of ACE-*c*, the percent apoptotic cells were assessed by Annexin V-FITC and propidium iodide staining, followed by flow cytometric analysis (Fig. 4A). It was observed that at concentrations of 40 and 80 μg/ml ACE-*c*, there was a significant increase in the percentage of cells undergoing apoptosis. Interestingly at 80 μg/ml ACE-*c *concentration, there was ~2.6-fold (*p *≤ 0.001) increase in the population of cells undergoing apoptosis compared to the untreated control cells.

Since intracellular Ca^2+ ^is a powerful activator of apoptosis [34,35], we studied the Ca^2+ ^signaling mechanism in cells treated with ACE-*c *to elucidate the cause of apoptosis. It was observed that after treatment of SiHa cells with various concentrations of ACE-*c *(0-80 μg/ml), there was a dose-dependent increase in the intracellular levels of calcium. It was noted that the calcium increase was maximal (~2.64-fold; *p *≤ 0.001) at the concentration of 80 μg/ml (Fig. 4B) compared to the control cells. Ionomycin was used as a positive control.

It is known that increase in intracellular calcium might be one of the factors responsible for disrupting the mitochondrial membrane potential resulting finally into cell apoptosis [36-39]. To measure the collapse of electrochemical gradient across the mitochondrial membrane, we stained the cells with JC-1 dye that aggregates into healthy mitochondria and fluoresces red. By confocal as well as flow cytometry assays, we observed that the cells exposed to ACE-*c *exhibited a dose-dependent decrease in JC-1 staining (Fig. 4C and 4D, respectively) compared to the untreated control cells. This indicated that there was a loss of mitochondrial membrane potential in cinnamon-treated cells, which approached the loss of potential observed after treating the cells with the positive control agent, FCCP (Fig. 4D). As clearly observed from the figure, cinnamon induced significant depolarization at 80 μg/ml ACE-*c *concentration wherein there was ~5-fold reduction in the ratio of red-green fluorescence intensity (p ≤ 0.001). Taken together, all these results suggested that cinnamon extract exhibited a potent antineoplastic effect in cervical cancer cells through increase in intracellular calcium flux as well as through loss of mitochondrial membrane potential, ultimately leading to apoptosis.

## Discussion

Dietary constituents may display promising chemopreventive and chemotherapeutic potential and thus ameliorate the side effects associated with conventional chemotherapy. Recently, more attention is being focused on complementary and alternative medicine (CAM) as an alternative therapeutic modality for treatment of cancer patients [2,3,9-13,40].

In the present study, we have reported the anti-cancer potential of cinnamon extract in vitro in a human cervical cancer cell line and have elucidated the possible underlying mechanism. The anti-tumor activity of cinnamon has been reported *in vitro *[21-23] as well as in *vivo *[10]; however, its role in cervical cancer remained to be elucidated. We found that the aqueous cinnamon extract significantly affected the growth rate of SiHa cells in a dose-dependent manner. This data was further supported by results from colony formation and soft agar assays, which demonstrated statistically significant reduction in the number of colonies in ACE-*c *treated cells compared to the untreated control cells. Thus, cinnamon could be proposed to be a promising candidate for restricting the growth of cervical cancer cells.

It is well known that metastasis, being one of the major causes of mortality in cancer, involves various steps such as cancer cell adhesion, invasion, and migration [41]. Thus, to examine the effect of cinnamon extract on migration of SiHa cells, wound healing assays were performed on untreated control and ACE-*c *treated cells. Interestingly, cinnamon reduced the migration of cancer cells in a significant manner, further strengthening its potential use as an anti-cancer drug in cervical cancer.

One of the key steps in the invasive progress of cancer cells is the degradation of extracellular matrix (ECM) proteins by a family of zinc-binding enzymes called as matrix metalloproteinases [42]. To elucidate the reason behind the poor migration of ACE-*c *treated cells, we tested the expression of MMP-2 (gelatinase) in control as well as cinnamon-treated cells. A significant decrease in the expression of MMP-2 was observed both at mRNA as well as protein levels in ACE-*c *treated cells that could be the reason for their reduced migration capability compared to the control cells. Thus, downregulation of MMP-2 expression by cinnamon could be regarded as a rational approach towards metastatic disease therapy in cervical cancer.

It is well-known that Her-2/Erb2, a transmembrane receptor protein with tyrosine kinase activity from EGF3-receptor family, is a critical marker of cervical and breast cancer. Moreover, it has been shown that Her2 overexpression is related with the invasion capacity of the tumor cells that is related partly with the up-regulation of MMP-2 and MMP-9 expression as well as proteolytic activity [32,33]. Interestingly, we found that cinnamon could effectively and significantly down-regulate the expression of Her-2 in SiHa cells. Thus, downregulation of Her-2 oncoprotein expression by cinnamon could be correlated with the reduction in the expression of MMP-2 protein. These leads could be explored in detail to further establish the antineoplastic activity of cinnamon in cervical cancer that would in turn emphasize the chemopreventive potential of natural products.

Apoptosis plays a key role in the regulation of normal tissue homeostasis and participates in the elimination of abnormal cells. Most of the antitumor drugs kill the cancer cells by stimulating the apoptotic pathway [43]. To test whether cinnamon could induce apoptosis in cervical cancer cell line SiHa, we carried out apoptosis studies. At an effective ACE-*c *concentration of 80 μg/ml, a significant proportion of cells were observed to undergo apoptosis compared to the control cells. To further elucidate the mechanism of apoptosis, we tested whether cinnamon could modulate calcium flux as the latter is known to be one of the major mediators of apoptosis.

Intracellular Ca^2+ ^trafficking is known to govern a number of vital cellular functions that affect cell survival. Cytosolic calcium, (Ca^2+^)_c_, is usually maintained at lower level (~100 nmol/L) compared to the extracellular concentration (~1 mmol/L). The cells regulate (Ca^2+^)_c _primarily by regulating the Ca^2+ ^trafficking across the plasma membrane and in and out of key organelles, such as the endoplasmic reticulum and the mitochondria [42]. The endoplasmic reticulum is the largest reservoir of Ca^2+ ^in normal cells whereas mitochondrial levels of Ca^2+ ^are quite low. But during apoptosis, mitochondria are known to accumulate Ca^2+^, especially when the (Ca^2+^)_c _level is high [35,37-39]. Increase in mitochondrial calcium, (Ca^2+^)_m_, induces apoptosis resulting into loss of Δψ_m_, expansion of the matrix, and the rupture of the outer mitochondrial membrane [36]. Interestingly, at 80 μg/ml concentration of cinnamon extract, there was a significant increase in the levels of intracellular calcium in SiHa cells that could result into their apoptosis.

Since cinnamon increased intracellular calcium levels as well as induced apoptosis in cervical cancer cells, we wanted to know the status of mitochondrial membrane potential in these cells. Moreover, mitochondria are known to accumulate Ca^2+ ^during apoptosis especially when the cytosolic calcium level is high [37-39,44]. Thus, we tested the Δψ_m _in cinnamon treated cells by using the fluorescent dye, JC-1 that aggregates into healthy mitochondria and fluoresces red. Upon mitochondrial collapse in apoptotic cells, JC-1 dye no longer accumulates and instead is distributed throughout the cell resulting into decrease in red fluorescence. In accordance with this, we found that ACE-*c *indeed disrupted the mitochondrial membrane potential as observed by decrease in the intensity of red fluorescence. FCCP, a drug known to disrupt the transmembrane potential of mitochondria [45], was used as a positive control. Thus, the increased intracellular calcium induced by ACE-c might be associated with the observed decline in mitochondrial membrane potential.

Dietary polyphenols have recently invited a great deal of attention owing to their chemopreventive properties [46-48]. They can modulate the process of carcinogenesis through several mechanisms. Interestingly, polyphenols seem to play dual roles (either protective or therapeutic) under different situations. For example, cinnamon polyphenols have been recently shown to play a protective role by attenuating the decline in mitochondrial membrane potential induced by ischemic injury in C6 glioma cells [49]. On the other hand, in our case, we observe that cinnamon extract, which contains a mixture of polyphenols together with cinnamaldehyde as the major bioactive component, plays a therapeutic role in cervical cancer cells through depolarization of the mitochondrial membrane potential resulting into cellular apoptosis. These natural products seem to work in a tightly regulated manner wherein they switch their roles either towards protective or therapeutic side depending upon either the amount of the drug being used or upon the cellular phenotype [50]. For example, resveratrol, another polyphenol, has also been shown to play a dual role, either protective [51-53] or therapeutic [54]. Based on all these observations, our data strongly implicates that cinnamon could be proposed to be a potent antineoplastic agent in cervical cancer wherein it could induce apoptosis through increase in calcium flux as well as through loss of mitochondrial membrane potential.

## Conclusion

The failure of conventional chemotherapy to reduce mortality invites attention towards new alternative approaches that would reduce morbidity as well as side effects conferred by conventional chemotherapy. Plants have played a significant role as a source of effective anticancer agents and 60% of currently used anti-cancer drugs are derived from natural sources such as plants, marine organisms and microorganisms [55,56]. Recently, a greater emphasis has been given towards the research involving complementary and alternative medicine in cancer management. Several studies have been conducted on herbs that possess anticancer properties and have been used as potent anticancer drugs [57]. The present work has addressed the antineoplastic potential of the spice cinnamon in cervical cancer. Cinnamon besides altering the growth kinetics of cells induces apoptosis through loss of mitochondrial membrane potential. Cinnamon reduces the expression of prognostic marker, Her-2, of cervical cancer that calls attention for further studies in this area. Collectively, these data suggest that cinnamon extract could be proposed to be a potent anticancer drug candidate in cervical cancer.

## Abbreviations

ACE-*c*: Aqueous Cinnamon Extract from *C. cassia*; CAM: Complementary and Alternative Medicine; Δψ_m_: Mitochondrial membrane potential; MMP-2: Matrix Metalloproteinase-2; MTT: 4,5-dimethylthiazol-2-yl-2,5-diphenylthiazolium bromide; FCCP: Carbonyl Cyanide *p*-(trifluoromethoxy) Phenylhydrazone; TMRE: Tetramethylrhodamine ethyl ester; Her-2: Human epithelial receptor 2; JC-1: 5,5',6,6'-tetrachloro-1,1',3,3'-tetraethyl enzamidazolocarbocyanin iodide

## Competing interests

The authors declare that they have no competing interests.

## Authors' contributions

RKG designed the study and drafted the manuscript. SJK and ASC have carried out all the experiments. SK had participated in cell growth analysis and RT-PCR experiments. SAS contributed to the FACS analysis. SC has helped in time-lapse study. All the authors read and approved the final version of the manuscript.

## Pre-publication history

The pre-publication history for this paper can be accessed here:

http://www.biomedcentral.com/1471-2407/10/210/prepub

## Supplementary Material

Additional file 1 **Biochemical Analysis and Cytotoxic Activity of Aqueous Cinnamon Extract (ACE-*c*)**. Data providing HPTLC analysis and polyphenol content of ACE-*c *as well as cytotoxic activity of the extract on SiHa cells. It includes supplementary figs. S1 and S2. Click here for file
