# Supplementary material for: Aqueous Cinnamon Extract (ACE-c) from the bark of Cinnamomum cassia causes apoptosis in human cervical cancer cell line (SiHa) through loss of mitochondrial membrane potential
Source: BMC Cancer. 2010 May 18;10:210. doi: 10.1186/1471-2407-10-210 (PMC2893107; doi:10.1186/1471-2407-10-210)

**Additional Files**

**Aqueous cinnamon extract (ACE-*c*) from the bark of *Cinnamomum* *cassia* causes apoptosis in human cervical cancer cell line (SiHa) through loss of Mitochondrial Membrane Potential**

**Supplementary Figures**

**Supplementary Figure S1.**

**Detection and quantification of cinnamaldehyde in aqueous cinnamon extract from *C.cassia* (ACE-*c*).** (A) The figure shows HPTLC chromatogram of standard mixture of piperine, cinnamaldehdye and eugenol (I) as well as ACE-*c* (II). (B) Calibration curve for quantification of total polyphenolic content in ACE-*c* by Folin-Ciocalteau method.

**Supplementary Figure S2.**

**Cytotoxic effect of ACE-*c* on human cervical cancer cells.** SiHa cells were treated with different concentrations (0-320µg/ml) of ACE-*c* for 24 h. The cell viability was measured by MTT assay.

**Supplementary Figure S1.**

**(A)**


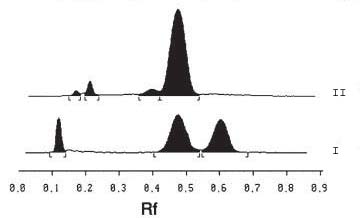


**(B)**


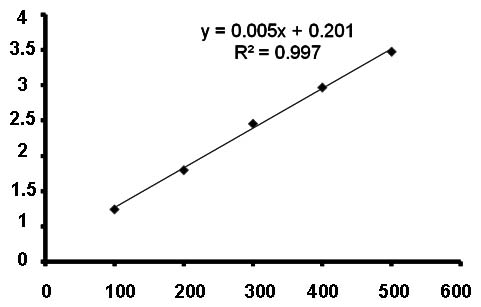


**Supplementary Figure S2.**


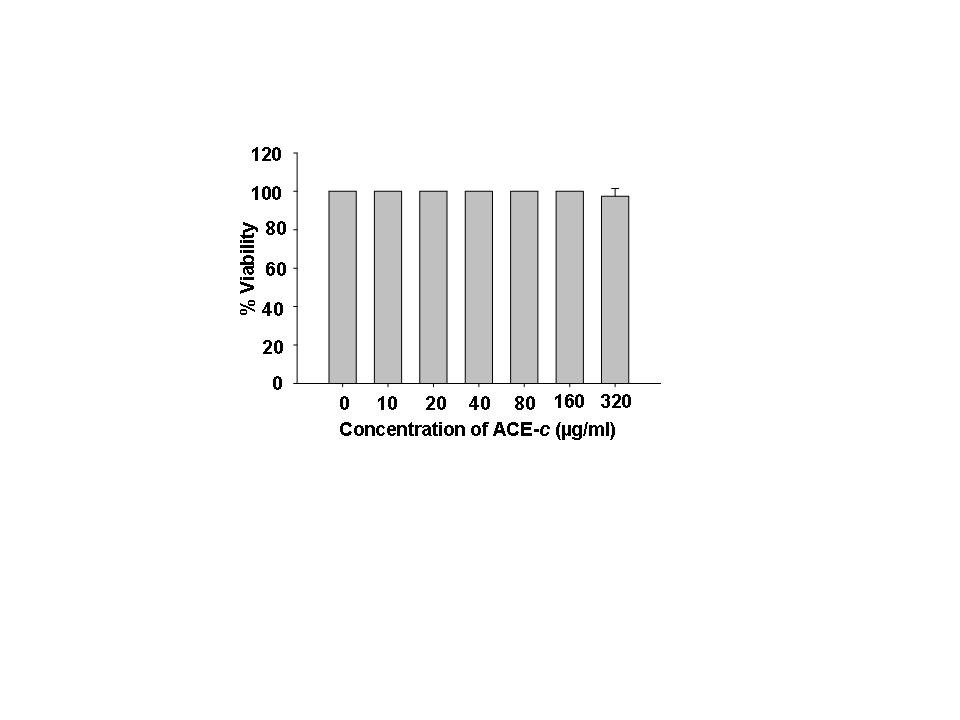

Supplement: Additional file 1 — Biochemical Analysis and Cytotoxic Activity of Aqueous Cinnamon Extract (ACE-c). Data providing HPTLC analysis and polyphenol content of ACE-c as well as cytotoxic activity of the extract on SiHa cells. It includes supplementary figs. S1 and S2. [file 1471-2407-10-210-S1.DOC]
